# Bacteriophages as Pathogens and Immune Modulators?

**DOI:** 10.1128/mBio.00868-13

**Published:** 2013-11-12

**Authors:** A. Lengeling, A. Mahajan, D. L. Gally

**Affiliations:** The Roslin Institute and Royal (Dick) School of Veterinary Studies, University of Edinburgh, Edinburgh, United Kingdom

## Abstract

While Shiga toxins (Stx) are key determinants of enterohemorrhagic *Escherichia coli* (EHEC) pathophysiology in humans, their dissemination to target organs following gastrointestinal EHEC infection is still poorly understood. Most types of Stx target cells with globotriaosylceramide (Gb3) receptors, which are expressed on endothelial cells. According to current theory, Stx is trafficked on the surface of peripheral blood cells, and transfer of toxin from these trafficking cells to endothelial cells results in microvascular damage to target organs, including the kidneys and brain. Inside the cell, Stx inhibits protein synthesis, resulting in cell death. Host “repair” responses can lead to microthrombus formation, erythrocyte damage, and reduced oxygen supply, potentially resulting in organ failure. A recent study [L. V. Bentancor et al., mBio 4(5):e00501-13, 2013, doi:10.1128/mBio.00501-13] indicates that another mechanism for Stx “dissemination” needs to be considered. Bentancor et al. demonstrated that high-pressure injection of a plasmid encoding the “prokaryotic” Stx2 sequence into mice can lead to mortality, with pathology indicative of Stx activity and antibody responses to Stx. While the plasmid levels and injection methodology were extreme, the study indicates that these sequences are potentially taken up into eukaryotic cells, transcribed, and translated, producing active Stx. Stx genes are present on integrated bacteriophage genomes in EHEC, and Stx-encoding phages are released following bacterial lysis in the gastrointestinal tract. We therefore need to consider whether bacteriophage sequences can be expressed in eukaryotic cells, what the wider implications are for our understanding of many “bacterial” diseases, and the possibility of developing novel interventions that target bacteriophages.

## EHEC AND Stx PATHOLOGY

Enterohemorrhagic *Escherichia coli* (EHEC) bacteria expressing different types of Shiga toxin (Stx) have emerged over the last 25 years as a serious threat to human health in many parts of the world. The toxins are important virulence factors of EHEC and are responsible for the more severe complications of EHEC-associated infections, such as hemorrhagic colitis and hemolytic-uremic syndrome (HUS) (1). The toxins have been classified into two main types (1 and 2) based on differences at the amino acid level, with more minor variation defining subclasses of Stx2. Both Stx1 and Stx2 are composed of a pentameric B subunit and a single A subunit. The B subunit defines cellular targeting to cells that express the globotriaosylceramide (Gb3, CD77) and to a lesser extent the globotetraosylceramide (Gb4) glycosphingolipid receptors on their surfaces. The combined B and A subunits enter the cell, and the presence of the B subunit is critical in membrane-associated trafficking before final release of the A subunit, which then acts in the endoplasmic reticulum to cleave 28S rRNA and inhibit protein synthesis (2, 3).

**FIG 1:**
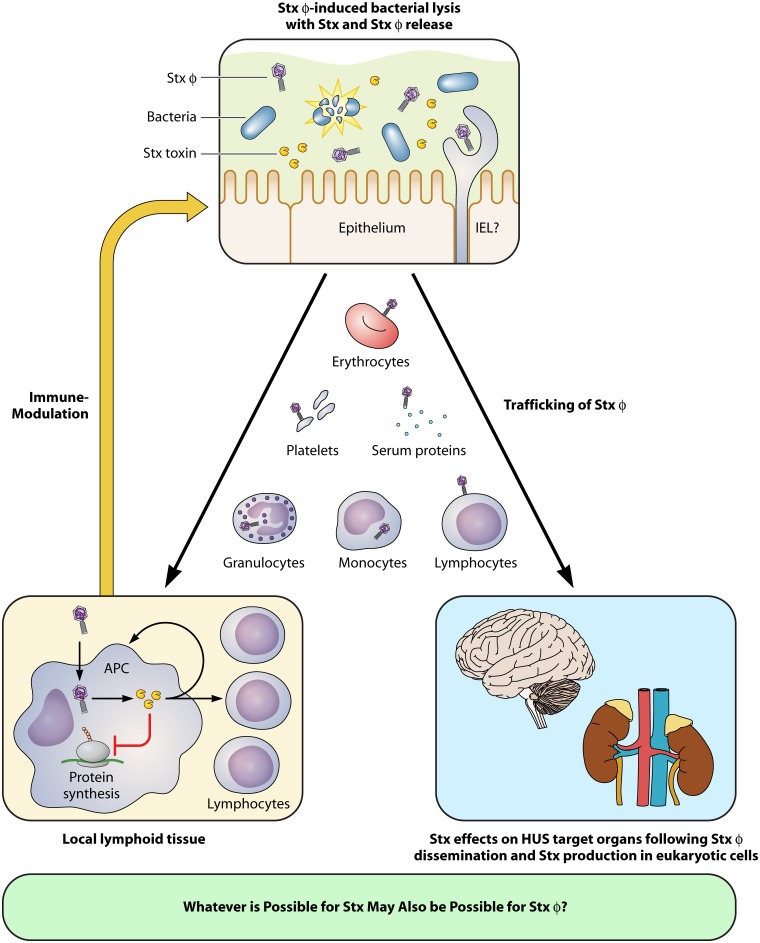
Schematic illustration of a proposed mechanism for Stx-encoding bacteriophage (Stxϕ) uptake into eukaryotic cells with subsequent Shiga toxin (Stx) production following human infection with EHEC. The top panel demonstrates lysis of EHEC in the gastrointestinal tract, with release of both Stx and Stxϕ. Stx or Stxϕ would have to translocate across the epithelial barrier, and this may involve transfer in or on eukaryotic cells, including intraepithelial lymphocytes (IELs). Systemic trafficking is shown for Stxϕ in the central section of the figure, but this also applies to Stx. The bottom-left panel shows the production of Stx in eukaryotic cells in the local lymph nodes following uptake of Stxϕ into cells, in particular monocytes and macrophages. The Stx produced can have an impact on local immune responses that feed back to impact the survival of the host bacterial population in the gastrointestinal tract. The right panel shows Stx production from Stxϕ in the main organs affected during HUS.

The main issue for human disease is the presence of the Gb3 receptor on endothelial cells that line our vasculature and the damage and subsequent repair responses in narrow capillaries of the microvasculature, in particular in the kidney and brain. This leads to thrombus formation with subsequent red blood cell destruction and prevention of blood/oxygen supply, which can in turn result in loss of tissue function and organ failure. Despite a sophisticated understanding of Stx activity, how the toxin actually traffics into systemic circulation from the bacteria in the gastrointestinal tract is still far from clear, and a number of mechanisms have been proposed (Fig. 1). The situation is made more complicated by the fact that the toxin genes are present on lysogenic bacteriophages and expression of the main Stx2 types requires induction of the bacterial SOS response with release of toxin occurring upon phage-induced lysis of the bacterial cell (4).

## EVIDENCE FOR A NEW VIEWPOINT ON Stx TRAFFICKING

In their recent publication, Bentancor and colleagues (5) provide evidence that a plasmid encompassing the prophage Stx2 genetic region can be lethal when injected systemically into mice, with evidence of Stx2 production and Stx2-induced pathology. The authors used a high-pressure delivery system to inject a large volume of plasmid DNA into the animals, which led to perfusion of the plasmid into different tissues throughout the body, in particular the liver. While the method of delivery is extreme, the results indicate that the “prokaryotic” plasmid is potentially taken up by eukaryotic cells, transcribed, and translated by the cellular machinery into active Stx. While there are a number of important possible caveats to this study (including the failure to use germfree mice, in which potentially transformable bacteria would be absent), the potential implications are far reaching. The possibility that Stx production occurs following uptake of Stx-encoding bacteriophages into certain types of eukaryotic cells should be tested, as should the wider possibility that bacteriophage gene expression is important to the etiology of several “bacterial” diseases. If proven to be true, this phenomenon opens up new opportunities for treatments that target the bacteriophage as well as the bacterium and key virulence factors.

In 1971, Merril et al. (6) demonstrated the expression of a lambdoid phage-encoded β-galactosidase in human fibroblasts. This was clear evidence that eukaryotic cells could take up and process phage particles, leading to production of a prokaryotic enzyme. While ground breaking, the pathogenic consequences of this startling work were, to our knowledge, never followed up. In the recent Bentancor study, Stx2 bacteriophages were not used, but the possibility remains that some level of Stx production may occur following uptake of Stx-encoding bacteriophages into specific eukaryotic cells, including dendritic cells, macrophages, and neutrophils. Further support for this idea comes from the use of bacteriophage lambda as a delivery vehicle for DNA vaccines (7). For vaccines, the antigen of interest is cloned into the bacteriophage genome and expression is driven by a strong eukaryotic promoter, but the point remains that once these bacteriophages are delivered systemically, then there is a mechanism by which the phage are taken up and the DNA exposed and then transcribed and translated, leading to sufficient antigen presentation to produce a decent adaptive immune response.

## CONSIDER BOTH BACTERIOPHAGE AND Stx TRAFFICKING IN THE ETIOLOGY OF EHEC-ASSOCIATED DISEASE

The findings of the Bentancor study necessitate a reevaluation of the current hypotheses regarding Stx dissemination. It is possible that these routes also apply to Stx-encoding bacteriophages, in which case bacteriophage uptake, followed by transcription and translation in eukaryotic cells, would lead to their systemic spread and to Stx production at distant sites (Fig. 1). Other hypothetical mechanisms of Stx dissemination include cell-based delivery of the toxin from the lumen of the intestine (where it is produced by the infecting bacteria) to target organs of HUS pathogenesis by peripheral blood cells, such as granulocytes, monocytes, platelets, lymphocytes, and erythrocytes (8–12). It has been suggested that these cells express low-affinity Gb3 receptors which allow Stx2 to bind, piggyback into the bloodstream, and traffic to endothelial cells of target organs that express high-affinity Gb3 receptors on their surface, including the kidneys and brain (9, 10). However, whether this really occurs in HUS patients is still heavily debated, since binding of Stx2 to these circulating blood cells could not always be reproduced *in vitro* and relevant animal models for investigation of *in vivo* Stx2 trafficking and delivery mechanisms are still absent. Other candidates for Stx2 trafficking include serum proteins, such as the serum amyloid P component (13), and lipoproteins that have Gb3 on their surface (14). The suggestion from the Bentancor research is that by acting as particles for transducing Stx-encoding sequences into eukaryotic cells, the bacteriophages themselves may also make a more direct contribution to HUS development as an alternative source of Stx2 production.

## ISSUES OF TRANSCRIPTION, TRANSLATION, AND ACTIVITY

In another recent study, Bentancor et al. provided preliminary data that indicate that the Stx2-encoding plasmid and phage can lead to expression of cytotoxic Stx2 in Vero cells (15). For Stx production in eukaryotic cells, the Stx2 sequences would need to be transcribed and translated. While there is potentially significant overlap between promoter and enhancer sequence requirements in prokaryotic and eukaryotic cells, the barriers to translation are much more significant. However, eukaryotic viruses are known to exploit noncanonical, cap-independent mechanisms, so this could apply to bacteriophages. It is also possible that the bacteriophage exploits gene expression and translation in mitochondria, given the close links between this organelle and the prokaryotic world. A further issue would be the site of Stx2 production, the potential impact of the toxin on the producing cell, and the details of whether and how the toxin is exported. While the Bentancor study demonstrated that very high levels of plasmid result in Stx-related pathology and anti-Stx responses, very low levels of Stx toxin can still have biological activity. As such, inefficient expression may still produce enough toxin to have an effect on host cells and potentially feed back to impact host-bacterium survival, providing selection pressure for the evolution of the expression away from the host bacteria.

## Stx IMMUNE MODULATION AND THE WIDER CONTEXT

Christian Menge’s work (16) has shown that Stx1 has a clear impact on B and T cell proliferation. Consequently, trafficking of Stx bacteriophages to local lymph nodes could lead to low levels of local toxin production, with subsequent immune-modulatory properties. Taking this one step further, it is interesting that bacteriophages associated with a number of pathogenic genera, such as *Clostridium* and *Staphylococcus*, are known to encode a number of toxins and immunomodulatory proteins and these are critical in defining the virulence of the bacterial pathogen in which they are hosted. We propose that the research community needs to consider the possible impact of released bacteriophages in health and disease and that these bacteriophage-encoded factors may be expressed away from the bacterium to modify immune responses that in the end benefit the bacteriophage-bacterium relationship. Bacteriophages are the most numerous replicating organisms on the planet and frequently come into contact with animals and humans. These are predominately prokaryotic DNA viruses, and perhaps we should not be surprised if they turn out to have a profound impact on their animal as well as bacterial hosts.
